# Evaluating the Role of Coenzyme Q10 in Migraine Therapy—A Narrative Review

**DOI:** 10.3390/antiox14030318

**Published:** 2025-03-06

**Authors:** Anna Fajkiel-Madajczyk, Michał Wiciński, Zuzanna Kurant, Józef Sławatycki, Maciej Słupski

**Affiliations:** 1 Department of Pharmacology and Therapeutics, Faculty of Medicine, Collegium Medicum in Bydgoszcz, Nicolaus Copernicus University, M. Curie Skłodowskiej 9, 85-094 Bydgoszcz, Poland; zuzanna.kurant@cm.umk.pl (Z.K.); jozef.slawatycki@cm.umk.pl (J.S.); 2 Department of Hepatobiliary and General Surgery, Faculty of Medicine, Collegium Medicum in Bydgoszcz, Nicolaus Copernicus University, M. Curie Skłodowskiej 9, 85-094 Bydgoszcz, Poland; maciej.slupski@cm.umk.pl

**Keywords:** coenzyme Q10, migraine

## Abstract

Migraine, with a prevalence of 14–15% in the world population, is one of the diseases that markedly reduce patients’ quality of life. Despite extensive therapeutic tools, the search for substances that may have potential therapeutic properties in migraine patients is still ongoing. Coenzyme Q10 (CoQ10), as a natural and potent antioxidant, appears to be a valuable adjunct in treating and preventing many conditions, such as cardiovascular, metabolic, autoimmune, or neurodegenerative diseases. This review aims to evaluate if CoQ10 can be a potential therapeutic agent in the treatment of migraine. Based on the studies discussed, CoQ10 may have applications in migraine therapy due to its potent anti-inflammatory and oxidative stress-reducing properties. Furthermore, by improving mitochondrial function, CoQ10 can contribute to the energy supply to brain cells, which is particularly important in migraine. Supplementation with CoQ10 in a wide range of doses has resulted in many therapeutic benefits in subjects, including a decrease in the frequency and duration of migraine attacks, a reduction in nausea, a lower maximum pain during an attack, and fewer days with migraine. Therefore, it seems that CoQ10 may be a relevant therapeutic supplement for the treatment and prevention of migraine.

## 1. Introduction

Migraine is among those diseases that significantly reduce the quality of patients’ lives. Despite the wide range of medications to treat migraine attacks (triptans, ergot derivatives, nonsteroidal anti-inflammatory drugs, 5-HT1F receptor agonist—lasmiditan [1]), it seems that the most critical issue for patients is the prevention of these attacks, for which monoclonal antibodies (erenumab, eptinezumab, fremanezumab, or galcanezumab) and gepants (atogepant, rimegepant) can be used [1,2]. Preventive methods also include physical activity [3], a balanced diet [4], and supplementation adapted to the body’s needs, e.g., prebiotics and probiotics [5]. It also appears that physiotherapy, with a focus on cognitive training and active exercise, can be a good non-pharmacological alternative to increase the pressure pain threshold in subjects with migraine [6]. Moreover, intensive mindfulness meditation seems to be an intriguing preventive method for migraine that, in studies, not only reduced the frequency and burden of the condition but also enhanced various psychological aspects of the participants and decreased the number of medications used [7]. An essential issue for migraine sufferers is avoiding certain foods known as “food triggers”, including alcohol, cheese, chocolate, coffee, cold cuts, citrus fruits, and excessive carbohydrates [8]. Despite extensive knowledge of the pathophysiology of this disease and the modern therapies available, the prevalence of migraine in the global population is 14–15%, and the disease mainly affects women [9,10].

Over the last few years, substances commonly found in products of plant or animal origin have gained particular importance in preventing and treating many diseases. One of these substances is coenzyme Q10 (CoQ10), a well-known natural antioxidant that protects cells from free radical damage [11]. Even though CoQ10 can be produced endogenously due to its limited absorption caused by genetic mutations and drugs, additional consumption may be a significant issue [12]. Despite the presence of CoQ10 in products such as eggs, dairy products, fish, meat, plant oils, nuts, vegetables, and fruits [13], many dietary supplements on the market promote increased absorption through their unique forms [12]. Since CoQ10 concentrations in the lungs, heart, spleen, liver, and kidneys noticeably decrease after age 20, additional supplementation of this substance seems physiologically justified [12]. Studies have shown that the use of CoQ10 can have a positive effect on reducing oxidative stress and cardiovascular mortality. In addition, using CoQ10 improves clinical outcomes in subjects undergoing coronary artery bypass surgery and reduces hypertension [14,15]. Interestingly, CoQ10, due to its properties, may positively affect autoimmune diseases associated with mitochondrial dysfunction, oxidative stress, and chronic inflammation [16]. It has been shown that CoQ10 may have health benefits not only for the elderly but also for children. Subsequent studies have shown that CoQ10 supplementation in younger individuals was also beneficial for acyl-CoA dehydrogenase deficiency [17], muscular dystrophy [18], migraine [19], attention deficit hyperactivity disorder (ADHD) [20], cardiomyopathy [21], and Friedreich’s ataxia [22]. Furthermore, it seems reasonable to use CoQ10 in neurodegenerative diseases with increased intracellular and mitochondrial production of reactive oxygen species (ROS), such as Parkinson’s disease [23].

Considering the widespread prevalence of migraine worldwide, this article summarizes the results of studies on the use of CoQ10 as a prevention or treatment of migraine.

## 2. Characteristics of CoQ10

**Figure 1:**
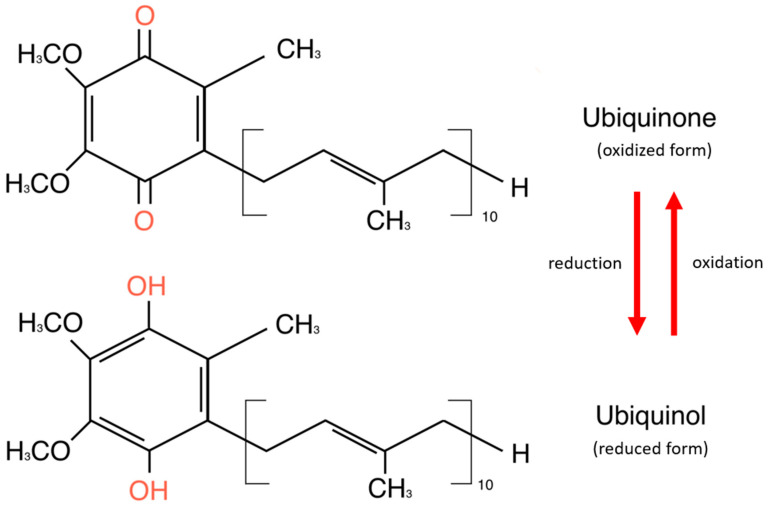
Chemical structures of ubiquinone and ubiquinol.

CoQ10 is a lipid-soluble molecule essential for adenosine triphosphate (ATP) production, as it is part of the mitochondrial electronic chain. It exists in two forms: oxidized and reduced [24]. The oxidized form of CoQ10, known as ubiquinone, is commonly found in dietary supplements and must be converted to its reduced form—ubiquinol—to function effectively in the body. The CoQ10 reservoir consists of cells with high energy requirements—the heart, liver, kidney, and pancreas. It is also detected in plasma in association with cholesterol-transporting lipoproteins [24]. Structurally, CoQ10 consists of a benzoquinone ring and 10 isoprenoid side-chain units attached to carbon 3 of the ring [25]. The benzoquinone ring of CoQ10 is a tyrosine derivative, while the side chain is derived from acetyl-CoA via the mevalonate pathway, which is common in synthesizing cholesterol and other lipids [26]. The basic structure of the coenzyme is conserved in all organisms, but the isoprenoid side chain is specific to each species [25]. Figure 1 shows the chemical structures of ubiquinone and ubiquinol.

Besides the previously mentioned antioxidant and anti-inflammatory effects of CoQ10, it is involved in regenerating vitamins C and E, among the potent antioxidants. Moreover, its contribution to the metabolism of pyrimidines, fatty acids, and mitochondrial uncoupling proteins as a cofactor of the dihydroorate dehydrogenase enzyme is worth mentioning [27]. Furthermore, CoQ10, as a potent lipid-soluble antioxidant, protects mitochondrial and extra-mitochondrial cell membranes such as the Golgi apparatus, lysosomes, peroxisomes, and endoplasmic reticulum from oxidative stress induced by free radicals [27].

In general, CoQ10 deficiency can be divided into primary, resulting from genetic defects in the multistep CoQ10 biosynthesis pathway, and secondary effects, associated with other diseases, such as cardiovascular disease, type II diabetes, or liver disease [27]. Because of its essential role in the human body, these deficiencies can have many health consequences and disrupt numerous cellular mechanisms in the organism.

While scientists still need to elucidate the many molecular mechanisms through which CoQ10 acts in the body, its supplementation is widely studied in several conditions. In recent years, research has shown that CoQ10 can benefit the male and female reproductive systems. Its antioxidant activity has been proven to increase fertility in men by, among other things, improving semen parameters and reducing oxidative stress on the testes [28]. In women, adequate supplementation with CoQ10 can enhance the quality of oocytes by improving mitochondrial function and reducing ROS levels [28]. Interestingly, the use of CoQ10 reduced levels of inflammatory markers such as tumor necrosis factor-α (TNF-α), high-sensitivity C-reactive protein (hs-CRP), and interleukin-6 (IL-6). In addition, a reduction in epithelium-related markers—vascular cell adhesion molecule-1 (VCAM-1) and E-selectin—was also noted [29]. Undoubtedly, CoQ10 may have a beneficial effect on the body’s lipid metabolism by inhibiting cyclic adenosine monophosphate (cAMP) degrading enzyme gene expression through the calcium/calmodulin-dependent protein kinase II—mitogen-activated protein kinase–extracellular signal-regulated kinase 1/2 (CaMKII-MEK1/2-ERK1/2) signaling pathway, which increases cAMP and activates the 5′AMP-activated protein kinase (AMPK) signaling pathway, which induces peroxisome proliferator-activated receptor alpha (PPARα) expression and inhibits adipogenesis [30]. Furthermore, the researchers suggest that CoQ10 may affect glucose metabolism by reducing the effects of oxidative stress on insulin secretion and, by blocking interleukin-1β (IL-1β), may inhibit glucose-stimulated insulin release from pancreatic islet cells [28]. CoQ10 may have potential neuroprotective properties by scavenging ROS, which protects neurons from oxidative stress [31,32]. Interestingly, CoQ10 may also have a beneficial effect on improving memory. Researchers have shown that oxidative stress and mitochondrial dysfunction lead to the formation of senile β-amyloid plaques, which increase acetylcholinesterase activity. CoQ10 effectively combats oxidative stress, leading to apoptosis and memory impairment, by activating glial cells [31,32]. Furthermore, researchers have shown that CoQ10 can promote wound repair. In a study on human keratinocyte HaCaT cells, CoQ10 increased caveolin-1 (Cav-1) localization in apical membrane domains of cells and Cav-1 content in membrane-rich fractions. Deprivation of this protein inhibited CoQ10-mediated wound repair and activation of phosphatidylinositol 3-kinase/protein kinase B (PI3K/Akt) signaling in HaCaT cells. It has been demonstrated that CoQ10 increases Cav-1 translocation to plasma membranes through the activation of the PI3K/Akt signaling pathway, which induces wound repair [33]. The beneficial protective effects of CoQ10 have also been reported in acute kidney injury. Its molecular mechanisms of action have been linked to the regulation of some critical genes, such as caspase-3, p53, and paraoxonase 1 (PON1), as well as signaling cascades, including the nuclear factor erythroid 2-related factor 2/heme oxygenase 1 (Nrf2/HO-1) pathway [34].

There is no doubt that the possible uses of CoQ10 discussed above represent only a tiny fraction of its therapeutic potential. Nevertheless, like any therapeutic agent, CoQ10 has several limitations. One is the bioavailability of exogenous CoQ10 due to its low water solubility and high molecular weight [35]. Studies have shown that CoQ10-containing preparations may show different bioavailability depending on the formulation used. A study comparing seven supplements containing 100 mg of CoQ10 proved that the soft-gel capsules containing ubiquinone or ubiquinol were the best absorbable formulation. Interestingly, the addition of antioxidants such as vitamin C to CoQ10 formulations and the matrix used to dissolve it significantly affected the bioavailability of CoQ10 [36]. In healthy adults, plasma CoQ10 levels typically range from 0.40 to 1.91 µmol/L. The maximum concentration (C_max_) of CoQ10 generally is achieved around 6 h, and it has an elimination half-life of about 33 h [37]. It may be particularly interesting to note that subjects receiving 100 mg of CoQ10 showed a higher or lower ability to achieve high blood CoQ10 concentrations. According to the researchers, unknown physiological factors significantly affect the bioavailability of CoQ10 [36]. Interestingly, other research indicates that CoQ10 is found in the blood almost exclusively as ubiquinol, even when ingested as ubiquinone [38]. The CoQ10 absorption pathways are similar to those of vitamin E. Initially, there is emulsification and micelle formation with fatty food components. This facilitates secretion from the pancreas and bile in the small intestine. The dose taken is also an important consideration, as well as taking CoQ10 with a meal, which may increase absorption [38]. To improve the bioavailability of CoQ10, formulations with reduced particle size and altered water solubility are being developed using methods such as complexation, solubilization, or reduction. In addition, modified-release formulations are also being developed [38].

Numerous clinical studies have confirmed the safety of CoQ10 supplementation in a wide range of conditions. The most common doses used were 200–300 mg/day for 3–6 months, but even with higher doses over a more extended period of therapy, no serious adverse effects were reported [39]. A double-blind, randomized, placebo-controlled clinical trial (Kaneka Q10) evaluated the safety of CoQ10 at daily doses of 300, 600, and 900 mg/day over 4 weeks. The most frequently reported adverse events included cold symptoms and gastrointestinal issues like abdominal pain and loose stools. However, these adverse effects were not dose-dependent and unrelated to the study [40]. Interestingly, other clinical trials have been conducted with a dose of 1200 mg/day of CoQ10, confirming the high safety profile of this substance [41].

## 3. Migraine Pathophysiology

The pathogenesis of migraine is multifactorial, and new factors underlying this disease are still being discovered. The more factors determining the disease, the greater the potential for drug action. At the same time, the large number of factors that determine migraine make drug control worse due to the activation of pathways other than those affected by medicaments. The fact that genetic and environmental factors are involved does not make it any easier either [42,43,44]. Moreover, most studies mention that the disorder results from interactions between these factors [45]. According to Grangeon et al., in one of the newest studies, 180 variants belong to complex molecular networks of ”pro-migraine” disorders [44].

Meningeal vasodilatation and inflammation are well known for their involvement in migraine. They are caused by the activation of disrupted neuro-vascular networks [46]. The trigeminovascular system (TVS) is a neuronal network supplying innervation for meningeal and brain vessels and dura mater [47]. The vital role in the correlation between migraine and TVS is a calcitonine gene-related peptide (CGRP), a 37-aminoacid neuropeptide. Its dominant subtypes expressed in trigeminal ganglion are the α- and β-isoforms [48]. It is secreted from 35 to 50% of neurons of the trigeminal ganglion, its central and peripheral terminals [48]. What follows from the above is that CGRP may act both peripherally—increasing the sensitivity of nociceptors, and in the central nervous system—enhancing sensory stimuli, thereby increasing pain perception [48]. A particular individual predisposition and subsequent dysregulation in this area leads to the disruption of these mechanisms and predisposes to a migraine. In addition, some authors confirmed that the concentration of CGRP during the migraine attack is higher [49]. This neuropeptide is also important in one more context—as a component of the trigeminovascular reflex, which involves the release of CGRP from nerve fibers in response to local cerebral vasoconstriction to dilate the vessels and prevent cerebral ischemia [50,51]. Some studies revealed that it has notable vasodilatory properties in cerebral circulation due to penetrating nerves releasing CGRP through the smooth muscles of vessels [52]. Also, the components of CGRP receptors exist on meningeal vessels [47].

The release of CGRP from peripheral nerves causes sensitization of trigeminal nerves and increased nitric oxide synthesis (NO). In summary, CGRP causes vasodilatation in two ways: endothelium-independent and endothelium-dependent vasodilatation [52]. In an endothelium-independent way, CGRP, due to its receptors, causes adenylate cyclase activation and cAMP increase, which activates protein kinase A (PKA), and this, in turn, phosphorylates ATP-dependent K+ channels, which leads to Ca^2+^ sequestration mechanisms and causes smooth muscle relaxation. In an endothelium-dependent way, due to G-related receptors, CGRP activates adylenate cyclase, which causes cAMP increase, activates PKA, and, as a result, activates endothelial NO synthase (eNOS), which occurs in NO production. NO diffuses into adjacent smooth muscle cells, activating guanylate cyclase, then leads to relaxation [52]. Vasodilatation, known for years as one of the causes of migraine attacks, is still mentioned in the newest research [53].

Even more interesting is that the dopaminergic neurons of a trigeminal nucleus caudalis (TNC) co-expressed CGRP. These neurons are projections from the A11 nucleus of the posterior hypothalamus. The hypothalamus is probably involved in the prodromal phase of migraine-attack initiation [54]. Imaging studies confirm this—more specifically, positron emission tomography, where increased blood flow in this area was demonstrated during the early prodromal phases of migraines [55]. Precursor symptoms like fatigue and yawning, along with the typical link between attacks and circadian and menstrual cycles, also suggest hypothalamus involvement [56]. Returning to the starting point is due to the dopaminergic projections from the hypothalamic A11 nucleus to the spinal trigeminal nucleus, widely known for their involvement in migraines.

The authors also mention other neuropeptides, such as substance P or neuropeptide Y, neurokinin A, pituitary adenylate cyclase-activating polypeptide, orexins, and nociceptives, which may play a role in neurogenic inflammation of the intracranial vasculature and peripheral and central sensitization of the trigeminal system [57,58,59].

Ion channel dysfunctions are also involved in migraine pathogenesis. Their proper functioning is essential in cell signaling, and their disorders in migraine patients are manifested by disruption of excitation–inhibition balance, neuronal excitability, and peripheral or central sensitization [60]. Dural afferent transducers such as acid-sensing ion channels (ASICs) and transient receptor potential cation channel (TRPV) subtypes are significant in migraines’ pathogenesis. They are engaged in sensing visual, auditory, mechanical, olfactory, auditory, thermal, and osmotic stimuli as environmental irritants. Their activation causes the influx of Ca^2+^ and Na^+^ ions and, as a result, leads to depolarization. The different subtypes of these receptors are involved in the transduction of different above-mentioned stimuli [60,61]. The potential stimulating events in the dura are mast cell degranulation secondary to stress, CGRP release, and nitroglycerin infusion, which are related to vasodilatation and sudden intracranial pressure changes. In addition, the expression of TRPV 1, for example, is proved in trigeminal neurons and brain regions engaged in migraine, and what is more, using its agonists provides increased calcium influx and, as a result, CGRP and substance P release, which initiates the cascade of neuroinflammation in migraine [62]. Authors also mention mutations of specific ion channels, such as TRESK—potassium channels encoded by gene KCNK18, which regulates the dorsal roots and trigeminal ganglia excitability; TRPM8, and others, which are linked with inherited migraine, which emphasize ion channels’ role in migraine [60,63]. Authors also often mention that mutations in genes coding ion channel proteins like CACNA1A, ATP1A2, and SCN1A, which encode voltage-gated calcium channels, are singled out in studies as an essential link in headache [64]. For example, a mutation in CACNA causes enhanced glutaminergic transmission because it allows the channels to open at more hyperpolarized membrane potentials than usual, which causes a more significant calcium influx to the terminal during the action potential. It leads to a disturbance of the balance in excitation/inhibition of the cortex into its stimulation, and cortical spreading depolarization (CSD) is thought to cause migraine aura [63]. CSD is a slowly propagated wave of depolarization, followed by a suppression of brain activity. It is characterized by a massive increase in K^+^, NO, and glutamate in the extracellular space. As a result, CSD stimulates the sensory neurons in the trigeminal ganglia, and K^+^, NO, and glutamate can activate nociceptive neurons in the meninges, and in some cases, involve changes in neural and vascular function, which results in changes associated with migraine aura, the strongest in the case of visual aura [64,65,66].

Studies also report on mutations in genes like SCN1A, which codes voltage-dependent Na^+^ channels, leading to inappropriate ion influx and showing an increased susceptibility to CSD. Mutation in ATP1A2, which codes an ATP-dependent transmembrane pump (Na+/K+ ATPase), likewise causes disorders of its functions, which, as a result, leads to an inappropriate intra- and extracellular K+ and Na+ gradient, which promotes excitatory cortical transmission and, thus, the initiation of CSD waves [44].

Leaving the topic of ion channels and returning to sensory stimuli in migraine, it turns out that people with migraine, especially those with photophobia during the attack, are more sensitive to some kinds of visual stimuli, like striped patterns [67]. Hyperexcitability, hyper-responsiveness, and a lack of habituation are theories explaining this phenomenon. Hyperexcitability theory suggests that visual areas may be overexcited due to lower thresholds [68]. Some authors propose that more accurately, it is a theory of hyper-responsiveness and lack of habituation [69,70]. The normal brain reaction to repeated stimuli is habituation, which reduces the brain’s response to this stimulation. Some authors reveal that migraineurs have a lack of habituation and, as a result, some visual stimuli or, for example, olfactory stimuli are triggers of migraine attacks. The habituation mechanism is very complex, and studies use VEP (visually evoked potential) to explain this mechanism. Still, we only wanted to highlight that habituation is also considered necessary in the pathogenesis of migraine [71].

There also exist studies that indicate an abnormal, higher-order thalamocortical communication pattern in migraine patients. The authors believe that a disturbance of thalamocortical concerted working leads to disorder in information flow and, as a result, deficit pain processing of migraine [72]. Other authors suggest that in some specific kinds of migraine-vestibular migraine, the functional connectivity between the thalamus and brain regions involved in pain are altered—reduced thalamic-pain and thalamic-vestibular pathways while exhibiting enhanced thalamic-visual pathway [73].

**Figure 2:**
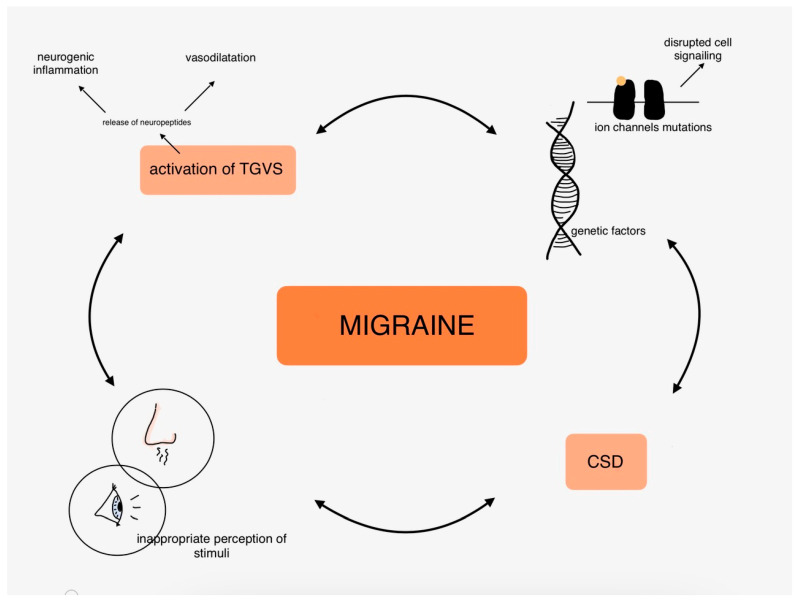
Pathophysiology of migraine in the human body.

The molecular mechanisms of migraines are covered in extensive articles, so we only touched on the most common hypotheses of migraine pathophysiology in this part. To summarize, it is worth underlining the role of neuroinflammation and neuropeptides, vasodilatation, ion channels, and genetic disorders highly related to inherited types of migraine and to remind that the pathogenesis of migraine is the complex connections between those mentioned above and many other factors. Figure 2 shows the complexity of migraine pathophysiology in the organism.

## 4. CoQ10 in Migraine Therapy

Based on the numerous therapeutic properties of CoQ10 and the complex pathogenesis of migraine, such supplementation may benefit migraine patients. Table 1 summarizes the available research findings.

Recent research highlights intriguing findings on the impact of CoQ10 in patients with migraines. Dahri et al. studied 45 subjects who took CoQ10 at 400 mg/day for three months in a randomized, double-blind clinical trial. They observed a decrease in migraine severity and a reduction in the frequency and duration of migraine attacks thanks to pain-alleviating properties [74]. The immune-boosting effects and antioxidant properties of CoQ10 result in lower levels of TNF-α and CGRP, both of which are recognized for their roles in migraine pathogenesis, as previously discussed in our review. CoQ10 appears to be a potent anti-inflammatory agent that can enhance the inflammatory aspect of migraines, making it crucial to consider as a protective supplement. The results of those studies outlined the ability of CoQ10 to reduce inflammatory markers through the regulation of gene expression [74].

In a prospective observational study, Guilbot et al. studied 132 participants who took CoQ10 at a daily dose of 100 mg, 100 mg feverfew, and 112.5 mg magnesium for three months. The results were a significant reduction in the number of days with migraine headaches, a decrease in the number of subjects with sensitivity to light and noise, and a reduction in the frequency of nausea, anxiety, and depression [75]. As is widely known, mitochondrial energy is deficient in the brain when dealing with migraines. CoQ10 is helpful in migraine prevention because of its crucial role in sustaining mitochondrial energy stores and stimulating the endothelial release of NO. Furthermore, the beneficial effects of the other components cannot be excluded in this study. Feverfew contains parthenolide, which is responsible for vascular smooth muscle relaxation. Magnesium accounts for ATP synthesis and function, glucose metabolism, and control of vascular tone. This study has shown that feverfew, magnesium, and CoQ10 administered together may have a synergistic effect as an anti-migraine treatment [75]. We handled this with great caution; while additional research is needed, our curiosity led us to seek further studies that might clarify the abovementioned points.

In a parallel-arm, double-blind, prospective multi-center study, Gaul et al. studied 130 subjects who were supplemented with 150 mg/day CoQ10, 400 md/day riboflavin, 600 mg/day magnesium, Migrant/Dolovent four capsules/day for three months. The results obtained in the study included a reduction in maximal pain intensity and a reduction in the HIT-6 questionnaire [76]. In contrast, there was no statistically significant difference in the decrease in days with migraine, which the researchers said could be related to the likely underpowering of the supplementation. This was the first clinical trial to use a combination of the ingredients mentioned above rather than using them as monotherapy. The researchers’ use of such a combination seems justified, as the deficit in the nutrients used plays an essential role in the pathophysiology of migraine. In addition, CoQ10, magnesium, and riboflavin play a key role in energy production in the mitochondria, which is widely known to be significantly associated with migraine [76].

Another study that again highlighted mitochondrial dysfunction in the pathogenesis of migraine was conducted by Hajihashemi et al., who studied 56 subjects for 8 weeks with 30 mg/day CoQ10 and 500 mg/day L-carnitine. They observed a decrease in HDR and a reduction in plasma lactate levels [80]. Lactate is a marker of mitochondrial metabolic disorders, and in this study, it was correlated with the primary severity of a headache. Its reduction may have a positive effect on the course of migraine. The studies discussed above highlight mitochondria’s critical role in migraine development. Due to their production of sufficient ATP and regulation of intracellular calcium levels, mitochondria are essential in neuronal function. Abnormalities associated with their functioning significantly affect energy metabolism and ion homeostasis in neurons, which increases the risk of developing migraine [80]. There is no doubt that CoQ10, as a factor protecting mitochondria from oxidative stress and being an essential component of energy metabolism, can significantly contribute to reducing migraine symptoms. However, due to the high-dose L-carnitine used in the study and its positive effects on mitochondria, all credit cannot be confidently attributed to CoQ10 alone.

In a parallel clinical trial with a control group and a comparative study, Yaghini et al. studied 72 children who took CoQ10 in a dose adjusted to body weight (x < 30 kg—30 mg/day; x > 30 kg—60 mg/day). Moreover, in a comparative study, they received amitryptiline for three months. In both groups, a decrease in duration, severity, and number of days with migraine was observed [77]. CoQ10 showed sound therapeutic effects with long-term use after three months of treatment and fewer adverse effects than amitriptyline, which has a more rapid response. Significantly, after three months, there were no differences between the group treated with CoQ10 and those treated with amitryptiline [77]. On the other hand, due to CoQ10 having less persistent side effects, it can be a good alternative for preventive migraine treatment, especially in long-term use.

Another clinical study by Dahri et al. studied 84 women aged 18–50 who were supplemented by 400 mg/day of CoQ10 for 12 weeks [78]. This study observed a significant increase in HDL-C levels and a decrease in BFP, which can be related to CoQ10’s impact on increased lipid oxidation in adipocytes and reduced lipogenesis. Moreover, the study showed a reduction in oxidative stress level markers—MDA and TAC [78], which is further evidence of the potent antioxidant properties of CoQ10.

In an open-label, single-arm, prospective, multicentre study, Vikelis et al. studied 113 adults who were administrated 20 mg of CoQ10, 281.25 mg magnesium, 4.8 mg vitamin B2, 150 mg feverfew, and 100 mg *Andrographis paniculata* for three months. Researchers observed a decrease in the severity and frequency of migraine headaches in a month [79]. Due to the supplementation of multiple components, it is difficult to predict what role CoQ10 alone played in this study. Nevertheless, the functions and effects of CoQ10 continue to be explored, which is why we chose to highlight this research.

Similar effects were achieved by researchers in a placebo-controlled, double-blinded crossover, add-on trial in which 120 subjects took 100 mg of CoQ10 for 224 days [19] and researchers in a placebo-controlled, double-blinded study with 42 subjects who were supplemented with CoQ10 at a dose of 300 mg/day for three months [81]. Both research groups, despite the use of different doses and different observation times, observed a reduction in the frequency of migraine attacks and a reduction in the severity and number of days with migraine headaches.

As mentioned in the introduction, migraine significantly impairs quality of life. Recurrent, continuous, and intense pain can cause migraine sufferers to withdraw from social life and daily activities. Studies have shown that compared to the healthy population, as many as 22.4% of migraine sufferers struggle with anxiety, and 25.9% of migraineurs suffer from depression [82]. As it turns out, the prevalence of depression is three times higher in migraine patients, but also patients with depression are more likely to suffer from migraine than the rest of the population [83]. Also, bipolar disease predisposes to migraine—almost 30–50% of patients with bipolar disorder could have both of these diseases [84]. In other sources, authors mention the relation between migraine anxiety disorders, in which the most potent connections are proved for generalized anxiety disorder, obsessive-compulsive disorder, and panic disorder [83,85]. Other diseases related to migraine are epilepsy, irritable bowel syndrome, *Helicobacter pylori* infection, celiac disease, and many others [84,86,87]. These are just a few examples that show how complex migraine therapy continues to be, due to the numerous co-morbidities and how many issues need to be considered to treat those affected individuals effectively. Furthermore, it is essential to emphasize that a deeper understanding of the CoQ10 mechanisms of action creates opportunities for treating various other migraine-related conditions, as it is a substance with multifaceted effects on the organism.

The discussed studies show that, despite using different doses and follow-up times, CoQ10 can be successfully used as a preventive and therapeutic agent in migraine. Due to the use of combination preparations in some studies, CoQ10 may act synergistically with other ingredients as an anti-migraine supplement. None of the above studies showed serious adverse effects caused by administered supplementation.

## 5. Future Perspectives

In light of the studies mentioned above, conducting research with larger subject groups in the future seems essential, as the studies discussed were carried out with relatively small groups. Furthermore, due to the use of CoQ10 in a wide range of doses, it appears crucial to develop the most effective dose and the minimum treatment time to achieve the best therapeutic effect. Due to the complexity of migraine pathophysiology, the topic should be approached more comprehensively, and all available tools should be used to combat this disease. Our suggestions for future research include using CoQ10 supplementation and non-pharmacological methods such as physiotherapy or meditation, as well as working on stress, which is often marginalized due to the fast pace of life.

**Figure 3:**
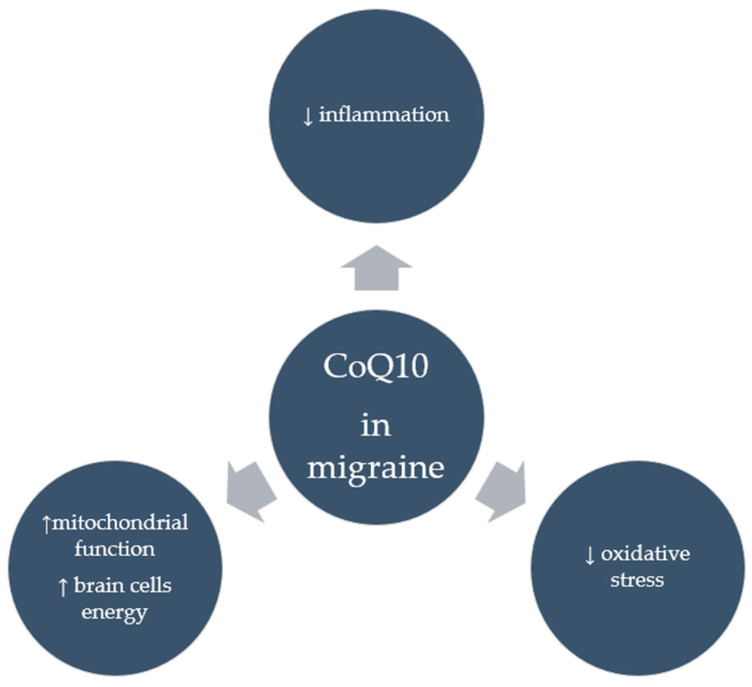
Suggested effects of CoQ10 in migraine.

Despite numerous clinical studies supporting the positive effects of CoQ10 in migraineurs, it appears that the mechanisms through which it acts in this group are not entirely clear. It seems that CoQ10’s potent antioxidant properties, whereby it neutralizes free radicals and reduces oxidative stress, in addition to the strong anti-inflammatory properties seen in the form of reduced inflammatory markers following CoQ10 supplementation, may account for its anti-migraine effects. Moreover, by improving mitochondrial function, CoQ10 can provide energy to brain cells, which is particularly important for migraine sufferers. Figure 3 shows the suggested effects of CoQ10 on migraine. However, research is needed to confirm these mechanisms.

## 6. Conclusions

The studies discussed in this review show that CoQ10 can significantly reduce the frequency and duration of migraine attacks. In addition, a reduced number of days with migraine and a decreased level of maximal pain during a migraine attack were also observed. Significantly, subjects experienced a notable reduction in comorbid symptoms, including nausea and sensitivity to light and noise. Additionally, subjects reported a marked improvement in their quality of life, along with decreased anxiety and depressive symptoms while using CoQ10. Also noteworthy are the laboratory results showing decreased inflammation and oxidative stress markers and improved lipid metabolism in migraine subjects who received CoQ10 supplementation. CoQ10 can be considered a valuable agent in the treatment of migraine and can be used in a wide range of doses due to its safety.

## Figures and Tables

**Table 1:** Summary of research findings on the use of CoQ10 in migraine.

Study	Characteristics of the Group and Duration of The Study	Daily Doses of CoQ10	Additional Substances	Effects on Symptoms and Lab/Instrumental Parameters
Dahri et al. (2019) [74]	45 participants 18–50 years old; 3 months.	400 mg/day	-	↓ duration, frequency and severity of migraine attacks; ↓ TNF-α; ↓ CGPR.
Guilbot et al. (2017) [75]	132 participants- adults aged 18–65 years old (“Intention to treat” population—68 people; “Per protocol” population—62 people); 3 months.	100 mg/day	Feverfew 100 mg/day; Magnesium 112.5 mg/day; Vitamin B6 1.4 mg.	↓ number of days with migraine headaches per month; ↓ number of subjects with sensitivity to lights and noise; ↓ frequency of nausea; ↓ anxiety and depression.
Gaul et al. (2015) [76]	130 adults aged 18–65 years old; 3 months.	150 mg/day	Riboflavin 400 mg/day; Magnesium 600 mg/day; Migravent/Dolovent 4 capsules/day.	↓ maximal pain intensity per migraine day; ↓ HIT-6 Questionnaire (headache impact test).
Yaghini et al. (2022) [77]	72 children aged 5–15 years old; 3 months.	<30 kg—30 mg/day >30 kg—60 mg/day	-	↓ number of days with migraine headaches per month; ↓ duration of headaches in 3 months; ↓ severity of headaches; improvement in subjects’ quality of life (QoL).
Dahri et al. (2023) [78]	84 adult women aged 18–50 years old; 12 weeks.	400 mg/day	-	↑ in HDL-C level ↓ body fat percentage (BFP) ↓ oxidative stress markers: malondialdehyde (MDA) and total antioxidant capacity (TAC)
Vikelis et al. (2020) [79]	113 adults; 3 months.	20 mg	1 or 2 tablets with 281.25 mg magnesium, 4.8 mg vitamin B2, 150 mg feverfew, 100 mg Andrographis paniculata	↓ mean migraine days; ↓ number of monthly days with peak migraine intensity of more than four (moderate/severe pain from 0 to 10 pain scale).
Slater et al. (2011) [19]	120 children and adolescents; 224 days.	100 mg/day	-	↓ number of days with migraine headaches per month; ↓ duration of migraine headaches.
Hajihashemi et al. (2018) [80]	56 adults aged 20–40 years old; 8 weeks.	30 mg/day	L-carnitine 500 mg/day	↓ HDR (headache diary results: duration of headache × frequency); ↓ Plasma lactate level.
Sándor et al. (2011) [81]	42 adults aged 18–65 years old; 3 months.	3 × 100 mg/day	-	↓ migraine attack frequency; ↓ headache- days; ↓ days with nausea.

↓ decrease; ↑ increase.
